# Effect of Copper Ion Concentration on Bacteria and Cells

**DOI:** 10.3390/ma12223798

**Published:** 2019-11-19

**Authors:** Lee Fowler, Håkan Engqvist, Caroline Öhman-Mägi

**Affiliations:** Division of Applied Material Science, Department of Engineering Sciences, The Ångström Laboratory, Uppsala University, Box 534, 751 21 Uppsala, Swedenhakan.engqvist@angstrom.uu.se (H.E.)

**Keywords:** copper ion, ion release, antibacterial, *S. epidermidis*, MC3T3

## Abstract

In the oral cavity, dental implants—most often made of commercially pure titanium—come in contact with bacteria, and antibacterial management has been researched extensively to improve patient care. With antibiotic resistance becoming increasingly prevalent, this has resulted in copper being investigated as an antibacterial element in alloys. In this study, the objective was to investigate the copper ion concentrations at which cyto-toxicity is avoided while bacterial inhibition is ensured, by comparing Cu ion effects on selected eukaryotes and prokaryotes. To determine relevant copper ion concentrations, ion release rates from copper and a 10 wt. % Cu Ti-alloy were investigated. Survival studies were performed on MC3T3 cells and *Staphylococcus epidermidis* bacteria, after exposure to Cu ions concentrations ranging from 9 × 10^−3^ to 9 × 10^−12^ g/mL. Cell survival increased from <10% to >90% after 24 h of exposure, by reducing Cu concentrations from 9 × 10^−5^ to 9 × 10^−6^ g/mL. Survival of bacteria also increased in the same range of Cu concentrations. The maximum bacteria growth was found at 9 × 10^−7^ g/mL, probably due to stress response. In conclusion, the minimum inhibitory concentrations of Cu ions for these prokaryotes and eukaryotes were found in the range from 9 × 10^−5^ to 9 × 10^−6^ g/mL. Interestingly, the Cu ion concentration correlating to the release rate of the 10 wt. % Cu alloy (9 × 10^−8^ g/mL) did not kill the bacteria, although this alloy has previously been found to be antibacterial. Further studies should investigate in depth the bacteria-killing mechanism of copper.

## 1. Introduction

Implant failure is a widespread problem, resulting from several etiological factors where, among others, stability during early implantation and excessive loading during service play a role in early and late, respectively, implant failure [1]. This is the case for dental and orthopedic implants, where additional factors such as age of patient, smoking, and diabetes increase failure risk in orthodontics [2], while, for example, osteolysis, fracture, and dislocation increase risk in orthopedics [3]. For both of these applications bacterial infection and subsequent failure have become increasingly prevalent. Two recent systematic reviews [4,5] estimated an average prevalence of infection related dental implant failures, ranging from 9.3–12.8% at the implant level and 18.5–19.8% at the subject level. This increase in failures is a result of biofilm producing bacteria causing localized inflammation and loss of the surrounding bone, resulting in implant loosening. In the case of dental implants, this phenomenon is known as peri-implantitis, a disease that has been linked to bacterial infection [6]. Case studies show that loss of alveolar bone is a possibility where implants might require removal several years after implantation [7]. Furthermore, this disease risks becoming much more prevalent as antibiotic resistance rises [8].

In order to replace antibiotics, antibacterial strategies such as the addition of silver to titanium, the exposure of TiO_2_ surfaces to UV light [9,10], the addition to antibacterial nanoparticles [11], novel surface design [12], and even thin film coatings [13] have all been investigated. With respect to bulk alloying and oxides, Ag has been found to be undesirable as a result of its efficacy being limited to environments of higher pH and elevated temperature levels [14], while, despite TiO_2_ forming naturally on all titanium surfaces when in contact with oxygen, the antibacterial effect can only be achieved after these surfaces are exposed to UV light [15]. In replacement of these, a recent alloying approach is to use copper in titanium as an antibacterial element. The results of such studies have noted an effective antibacterial affinity by these alloys in the range from 3 wt. % to 10 wt. % of Cu [16,17,18]. Despite the promising results, not much is known concerning the mechanisms by which Ti-Cu alloys cause bacterial death, but Cu ions are believed to play a role in killing at a distance from the surface [19]. The cause of the distal antibacterial effect has been postulated to be a result of Cu ions producing reactive oxygen species (ROS), which rupture the cell wall of bacteria [20]. This has been said to occur according to the Fenton chemistry reaction (Equation (1)), where Cu reacts with hydrogen peroxide to produce hydroxyl products [19]. In addition, Cu ions have shown the ability to prohibit expression of biofilm forming enzymes such as *agr* and *sae* in *Staphylococcus aureus* [21].

(1) $$Cu^{+}+H_{2}O_{2}\;\to \;Cu^{2+}+OH^{-}+OH^{.}$$

While these bacteria might be vulnerable to ROS, they have the ability to scavenge H_2_O_2_, thus limiting hydroxyl production and surviving copper-induced stresses [21]. Correspondingly, mammalian tissue may suffer damage from excess copper exposure, while for copper deficient individuals, differentiation of blood cells may be hindered and patient health could suffer as a result [22]. For this reason, the World Health Organization has stated a copper limit of 2–3 mg/day [23]. Therefore, in this study the aim was to find a suitable concentration of Cu ion release from materials to limit bacterial resistance and reduce biofilm formation, while at the same time ensuring that mammalian cell toxicity is avoided. 

## 2. Materials and Methods

### 2.1. Inductively Coupled Plasma (ICP) Spectrometry Investigation

An ICP study was performed to determine the ion release from a known antibacterial Ti-Cu alloy, i.e., 10 wt. % Cu, taken from a previous study [24], as well as an almost pure copper alloy (99.5 wt. % Cu, JX Nippon Mining and Metals Corporation, Tokyo, Japan) to define a relevant upper range of Cu ion concentrations to investigate. Three tokens of 5 mm in diameter (heights reported in the Result section) of each material were immersed in a 2% HNO_3_ solution for a period of 24 h at 37 °C. The surface area to volume ratio of water was set at 1.5 cm^2^/mL, according to ISO Standards 10993-12:2002 and 10993-15:2000 [25,26]. Samples placed in Falcon tubes were covered with Parafilm^®^ M (Sigma Aldrich) while in the oven, to prevent evaporation. The concentrations of Cu were determined for each sample, using inductively coupled plasma optical emission spectroscopy ICP-OES (Avio 200, PerkinElmer Inc., Shelton, CT, USA) with a detection limit of 0.4 μg/L. Statistical analysis was automatically done in Synergistix for inductively coupled plasma optical emission spectroscopy (ICP-OES, PerkinElmer, version 2) and tabulated. The relative standard deviation (RSD) value, reported as a percentage, is used in ICP-OES studies to describe the precision of the measurements that are performed on a sample.

### 2.2. Experimental Procedure

Based on the findings from the ion release study, copper chloride dihydrate salt (Sigma Aldrich, Steinheim am Albuch, Germany) in MilliQ water (QPAK 2, Millipore) was used to create solutions with different copper ion concentrations, ranging from 0.009 ng/mL to 9 mg/mL (see Table 1). Pure MilliQ water was used as a negative control, while solutions of 2.5% DMSO in media (for cells, i.e., MC3T3) and 11% ethanol in MilliQ water (for bacteria, i.e., *Staphylococcus epidermidis*) were used as positive controls. To investigate the minimum inhibitory concentration (MIC) of Cu ions, as well as cell viability, consecutive dilutions were used (see Table 1). All solutions were sterilized by filtering with a 0.2 μm sterile filter and then placed under a UV lamp at close proximity for 30 min.

### 2.3. Bacteria Luminescence

A MIC test was done at various concentrations of Cu ions (Table 1) to study the influence of the Cu ions on the bacteria. The XEN43 *Staphylococcus epidermidis* bacteria (Perkin Elmer) were used in this study. This type of bacteria is bioengineered to undergo luminescence through the *luxABCDE* gene in a genetically modified strain called *S. epidermidis* 1457 [27,28]. To ensure the test validity and minimum variance in the bacteria grown in broth, a single colony-forming unit (CFU) was chosen from freshly grown bacteria on bacteriological agar (Sigma Aldrich). Then, 120 μL of Tryptic Soy Broth (TSB, Sigma Aldrich, Stockholm, Sweden) was added to white 96-well plates (NUNC, ThermoScientific). This was followed by the addition of 30 μL (3 × 10^7^ CFU/mL) of bacterial inoculum per well, which had been incubated a priori overnight at 37 °C, and tested to have optical density of 10^9^ CFU/mL [15] using a UV spectrophotometer with wavelength of 600 nm (Shimadzu Model, UV 1800, Tokyo, Japan). The luminescence was recorded to ensure that all wells contained similar amounts of bacteria using a Hidex Plate CHAMELEON V (425-106 Multilabel counter, Hidex Oy, Turku, Finland) equipped with Mikrowin software (version 4.34, Labsis Laborsysteme GmbH, Neunkirchen-Seelscheid, Germany). This was followed by the addition of 50 μL of either one of the solutions with varying Cu ion concentrations, or one of the control solutions (negative or positive) in replicates of 4. Thereafter, luminescence data was recorded for all sample wells, at hourly intervals for 7 h. The luminescence of the sample solutions was compared to the luminescence of the negative control (MilliQ water) using Equation (2) for antibacterial rate [18] at 5 h.

(2) $$R=\frac{N_{control}-N_{sample}}{N_{control}}\times 100$$

### 2.4. Bacteria CFU Study

The bacteria in this study were also evaluated using conventional plate counting techniques as recommended by the European committee on antibacterial susceptibility testing (EUCAST) [29]. The XEN43 bacteria were grown overnight in 10 mL (Sigma Aldrich, Stockholm, Sweden) at 37 °C and, thereafter, adjusted to optical density of 10^9^ CFU/mL [15] at 600 nm. Then, 30 μL of bacterial inoculum was added to each well in clear 96-well microtiter plates (VWR), followed by 120 μL of TSB (Sigma Aldrich, Stockholm, Sweden). Then, the respective solutions (see above), in replicates of 4, were added. The exposure of the bacteria to the solutions was done at 37 °C for 5 h. After the exposure, 100 μL of the bacterial solutions from each well was inactivated in 900 μL of Dey-Engley neutralizing broth (Remel^TM^, Thermo Scientific, Lenexa, KS, USA), and left for 5 min. After this initial dilution, the inactivated bacterial solution was diluted by pipetting 100 μL of the neutralizing broth into 900 μL of peptone water (Sigma Aldrich, Stockholm, Sweden), followed by consecutive dilutions of 100 μL into a series of sterile 2 mL Eppendorf tubes, containing 900 μL of peptone water each. Then, 800 μL of the peptone water with bacterial dilutions were plated using the pour plate method onto bacteriological agar (Sigma Aldrich, Stockholm, Sweden) mixed with TSB (Sigma Aldrich, Stockholm, Sweden) and allowed to grow for 24 h before being counted manually. Only petri dishes with bacterial colonies in the range from 30 to 300 were included in the analysis, since below 30 the counting statistics are poor and above 300 the bacteria can grow too close together. The test was repeated thrice for statistical purposes. The CFU for the samples was determined according to Equation (3).

(3) $$CFU=\frac{counted\;colonies\;\times \;dilution\;factor}{volume\;of\;culture\;plated\;into\;petri\;dish}$$

### 2.5. Cell Study

MC3T3 cells (Murine calvarial osteoblasts, ATCC) were used to investigate cell viability in the same copper ion solutions as the bacteria were studied. The MC3T3, which is an osteoblast precursor cell line derived from mouse calvaria, was chosen since the thought application of Ti-Cu alloys is orthopedic and dental implants, which are in direct contact with bone. 

The cell study was done with the Prestoblue viability assay (Invitrogen, Carlsbad, CA, USA).

Cells were grown in flasks at 37 °C in 5% CO_2_ atmosphere until reaching 8 × 10^5^ cells/mL. The cell culture media contained 1% Penicillin-Streptomycin (ThermoFisher, Thermo Fisher Scientific Inc., Gothenburg, Sweden), 10% fetal bovine serum (Gibco, Life technologies, Thermo Fisher Scientific Inc., Gothenburg, Sweden), and the rest was Dulbecco’s Modified Eagle Medium (DMEM, Gibco, Life technologies). Thereafter, cells were detached from the flasks using TrypLE Express Enzyme with Phenol red (Gibco, ThermoFisher, Thermo Fisher Scientific Inc., Gothenburg, Sweden).

In clear 96-well plates, (tissue culture, VWR) 8000 cells (based on standard curves) were plated in each well, and 150 μL of cell culture media was added to each well. The test proceeded by first allowing overnight attachment of the cells to reach confluence in the wells of close to 80% or more. Then 50 μL of the Cu ions solutions (Table 1), as well as a negative control (MilliQ water) and a positive control (2.5% DMSO in media [30,31,32,33]), was added in replicates of 4, and the cells were left for 5 h of exposure to the solutions, followed by 24 h of exposure. A viability assay with PrestoBlue (Invitrogen, Thermo Fisher Scientific Inc., Gothenburg, Sweden), a nontoxic solution that contains resazurin, a molecule that is reduced by mitochondrial activity in living cells to a fluorescent molecule and correlates to the number of living cells in the test volume, was used to evaluate cell survival. After 5 h and before the exposure to Prestoblue, the Cu ion solution was removed and cells were washed in phosphate buffer saline (PBS). In the next step, a 10% solution of PrestoBlue and media was mixed and the cells were exposed to 150 μL of the mixture for 1 h. For counting of fluorescence, 100 μL of the solution from each well was plated into black fluorescence plates and quantified in a plate reader (infinite, M200, TECAN trading AG, Männedorf, Switzerland) at wavelengths of 560 nm for excitation and 590 nm for emission (monochromatic setting). After exposure to Prestoblue, the cells were washed again in PBS, before the same Cu ion solution (or control solution) was added to the well for the 24-h exposure. Thereafter, the steps of cleaning in PBS, exposure to PrestoBlue, and counting of fluorescence were repeated.

### 2.6. Statistical Analysis

Statistical analysis was done in Origin (2018b, OriginLab Corp., USA) and Minitab 15 (Coventry, U.K.) for parametric and nonparametric analyses, respectively. The Brown–Forsythe homogeneity test (with *p* = 0.005) was performed on each data set to determine the suitability for parametric or nonparametric analysis. For the parametric analyses, a one-way ANOVA followed by a Tukey honest significance difference (HSD) post hoc test was used, while for the nonparametric analyses, the Kruskal–Wallis test was done to compare the various concentrations of Cu ions, from 9 × 10^−12^ to 9 × 10^−3^ g/mL, and the negative and positive controls. A statistical significance setting of *p* < 0.05 was used in both cases. Mean values and standard deviations are indicated in the plots.

## 3. Results

### 3.1. Inductively Coupled Plasma (ICP) Spectrometry Study for Cu

The tested alloys in 2% HNO_3_ gave readings as tabulated in Table 2 and the height of each token is also reported. The Cu ions release for the TiCu-10 alloys was on average 0.07 ± 0.004 mg/L, while, as expected, the released from the 99.5% Cu samples was significantly higher (*p* < 0.001) with an average of 2.75 ± 0.136 mg/L. 

### 3.2. Bacteria Luminescence

**Figure 1:**
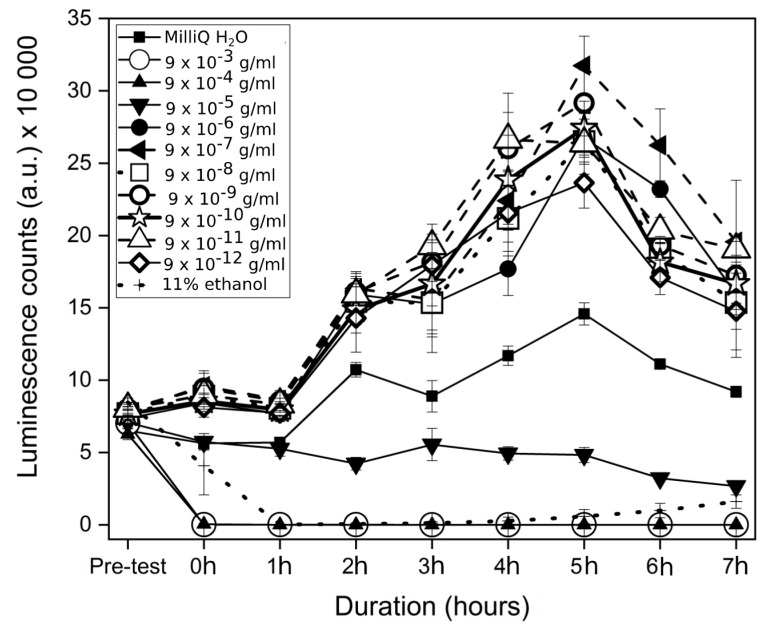
Luminescent counts (mean ± SD) of XEN43 bacteria in the investigated samples over the 7 h testing time.

**Figure 2:**
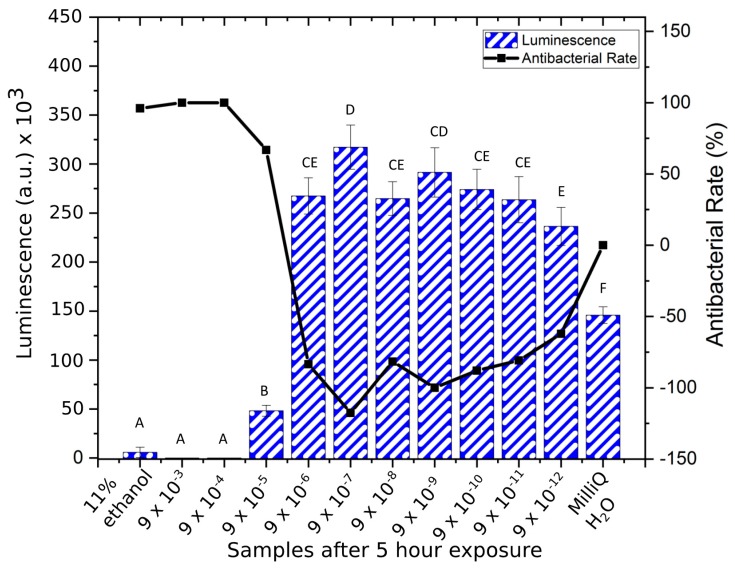
Luminescence counts (mean ± SD) of XEN43 bacteria after 5 h for all sample solutions, the negative (MilliQ) and positive (11% ethanol) controls, and the corresponding antibacterial rate. Brown–Forsythe: F (1, 11) = 1.48, *p* = 0.1802. One-way ANOVA: F = 238.9 (*p* < 0.001). Note: Samples indicated with the same letter are non-significantly different, e.g., “A” denotes that samples indicated with “A” are non-significantly different (*p* > 0.05). This also applies to samples indicated with “B”, “C”, “D”, “E”, and “F”. In addition, e.g., “CE” denotes non-significantly different to samples indicated with “C” and “E”. Conversely, samples indicated with differing letters, denotes statistically significantly different (*p* < 0.05).

The investigation into the bacterial response to Cu ions showed remarkable susceptibility by *Staphylococcus epidermidis* at high concentrations, where at concentrations of 9 × 10^−3^ and 9 × 10^−4^ g/mL, the luminescent signal from the bacteria immediately disappeared after the addition of Cu ions (Figure 1), similar to the results for the positive control (11% ethanol). However, the positive control killed all the bacteria over the first hour, while the 9 × 10^−3^ g/mL to 9 × 10^−4^ g/mL samples killed bacteria faster. The maximum growth of the bacteria was seen at concentrations of 9 × 10^−7^ g/mL while at lower concentrations, from 9 × 10^−8^ g/mL to 9 × 10^−12^ g/mL, samples gave a lower luminescence signal. The negative control (MilliQ H_2_O) showed lower growth rate than the 9 × 10^−7^ g/mL to 9 × 10^−12^ g/mL solutions. The fifth hour indicated the maximum luminescence for the bacteria and the bacterial count was reduced overall after this time point. At the fifth hour, the positive control (11% ethanol), 9 × 10^−3^, and 9 × 10^−4^ g/mL were not statistically different in luminescence counts (*p* > 0.70), and the antibacterial rate was highest for these (Figure 2). The samples 9 × 10^−6^ g/mL and 9 × 10^−8^ g/mL to 9 × 10^−12^ g/mL were not statistically different (*p* > 0.09), and showed bacterial population growth (negative antibacterial rate). The 9 × 10^−5^ g/mL and negative control samples were statistically different to each other (*p* < 0.01) and to all other samples (*p* < 0.05) with an antibacterial rate of 67% for the former mentioned sample, after 5 h of exposure.

### 3.3. CFU Counts for XEN43

**Figure 3:**
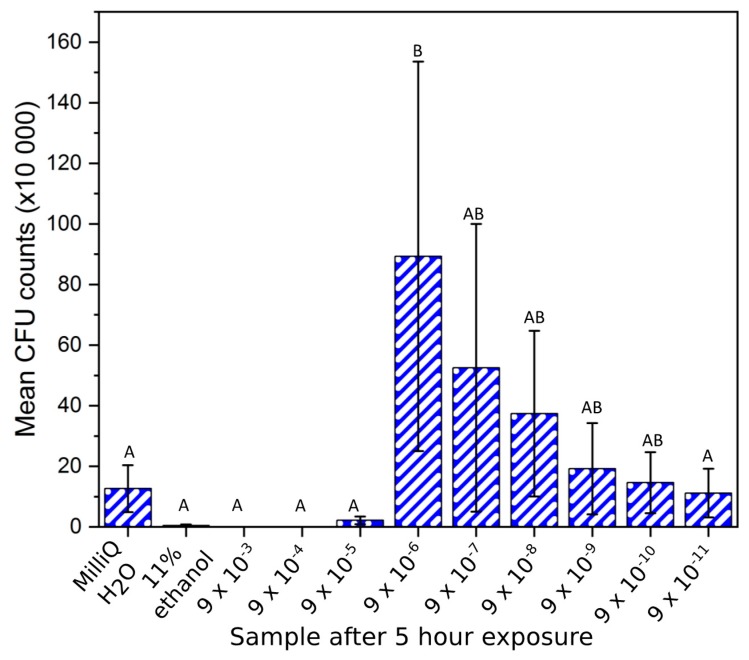
Colony-forming unit (CFU) counts (mean ± SD) of XEN43 bacteria after 5 h for all sample solutions, the negative (MilliQ) and positive (11% ethanol) controls. Brown–Forsythe: F (1, 10) = 1.78, *p* = 0.125. One-way ANOVA: F = 3.39 (*p* = 0.008). Note: Samples indicated with the same letter are non-significantly different, e.g., “A” denotes that samples indicated with “A” are non-significantly different (*p* > 0.05). This also applies to samples indicated with “B. In addition, e.g., “AB” denotes non-significantly different to samples indicated with “A” and “B”. Conversely, samples indicated with differing letters, denotes statistically significantly different (*p* < 0.05).

**Figure 4:**
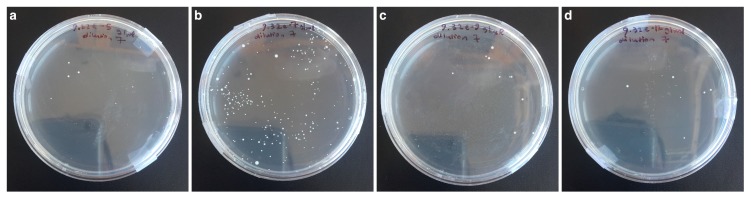
Example images of the bacteria count test after 5 h for samples: (**a**) 9 × 10^−5^ g/mL Cu ions, (**b**) 9 × 10^−7^ g/mL Cu ions, (**c**) 9 × 10^−9^ g/mL Cu ions, (**d**) 9 × 10^−12^ g/mL Cu ions. All after 7 iterative, 10-fold dilutions.

The *Staphylococcus epidermidis* bacteria plated in bacteriological agar gave similar results to the luminescence study. A high antibacterial ability was found for the positive control and solutions from 9 × 10^−3^ g/mL to 9 × 10^−5^ g/mL, relative to the negative control (Figure 3). However CFU counts were not statistically different among these samples or to the 9 × 10^−11^ g/mL sample (*p* > 0.99). The positive and negative controls and concentrations from 9 × 10^−3^ g/mL to 9 × 10^−5^ g/mL were statistically different to the 9 × 10^−6^ g/mL sample (*p* < 0.05). The 9 × 10^−6^ g/mL solution gave the highest bacterial count of all solutions after 5 h. However, due to the large variance, it was not statistically different to samples 9 × 10^−7^ g/mL to 9 × 10^−10^ g/mL. Samples 9 × 10^−7^ g/mL to 9 × 10^−11^ g/mL showed a decline in the CFU count for the bacteria (Figure 4), but these samples were not statistically different to each other or any of the other samples studied (*p* > 0.06). 

### 3.4. Cell Study 

**Figure 5:**
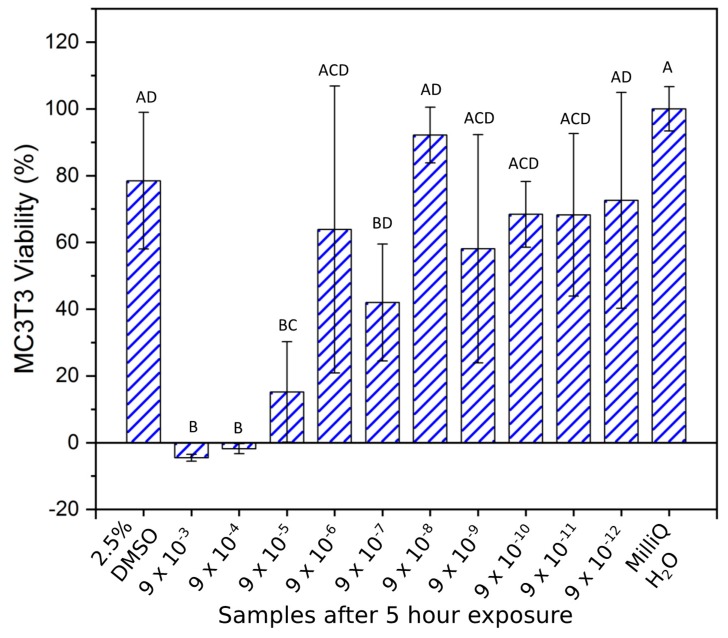
MC3T3 cell viability (mean ± SD) investigation after 5 h of Cu ion exposure. Brown–Forsythe: F (1, 10) = 1.54, *p* = 0.161. One-way ANOVA: F = 9.92 (*p* < 0.001). Note: Samples indicated with the same letter are non-significantly different, e.g., “A” denotes that samples indicated with “A” are non-significantly different (*p* > 0.05). This also applies to samples indicated with “B”, “C”, and “D”. In addition, e.g., “AD” denotes non-significantly different to samples indicated with “A” and “D”. This also applies to samples indicated with “BD”. Samples indicated with “ACD” denotes non-significantly different to samples indicated with “A”, “C”, and “D”. Conversely, samples indicated with differing letters denote statistically significantly different (*p* < 0.05).

At 5 h (Figure 5) the toxicity of the copper ion solutions from 9 × 10^−3^ to 9 × 10^−5^ g/mL was significantly greater (*p* = 0.01) than the positive control (2.5% DMSO). At concentrations of 9 × 10^−6^ g/mL and lower the viability significantly increased, compared to the 9 × 10^−5^ g/mL solution (*p* > 0.06). Compared to the negative control all concentrations of Cu ions of 9 × 10^−6^ g/mL and lower, excluding the 9 × 10^−7^ g/mL sample (*p* = 0.03), were not significantly different (*p* > 0.48) in terms of MC3T3 cell survival. 

**Figure 6:**
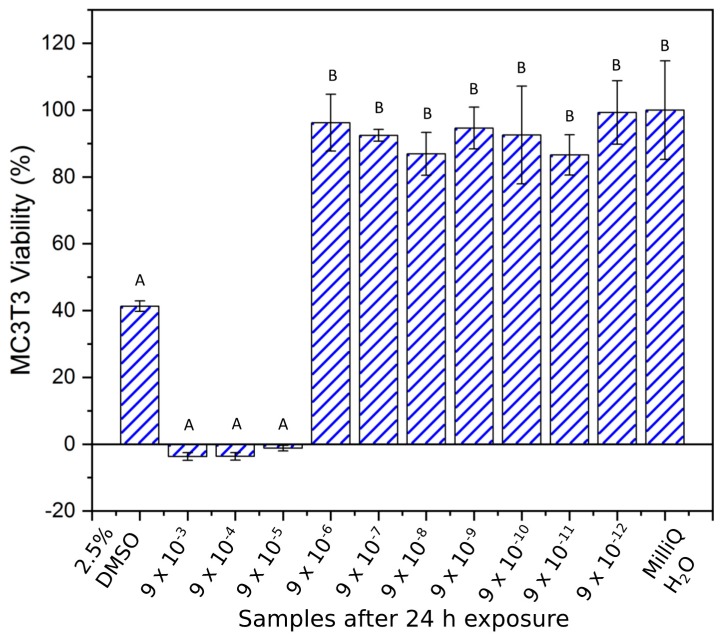
MC3T3 cell viability (mean ± SD) investigation after 24 h of Cu ion exposure. Brown–Forsythe: F (1, 10) = 3.22, *p* = 0.004. Nonparametric Kruskal–Wallis test showed significant differences among groups (H = 36.53, df = 11, *p* < 0.001). Note: Samples indicated with the same letter are non-significantly different, e.g., “A” denotes that samples indicated with “A” are non-significantly different (*p* > 0.05). This also applies to samples indicated with “B”. Conversely, samples indicated with differing letters denote statistically significantly different (*p* < 0.05).

The 24-h viability test showed similar results to the samples at 5 h, but in this case the Brown–Forsythe homogeneity assumption was violated and the nonparametric Kruskal–Wallis test was used to determine significant differences among the samples. No statistical difference (*p* < 0.05) was found among concentrations from 9 × 10^−3^ to 9 × 10^−5^ g/mL compared to the positive control, where the viability of MC3T3 cells was lower than 10%. These samples were statistically different (*p* < 0.05) to the samples from 9 × 10^−6^ g/mL to 9 × 10^−12^ g/mL, and the negative control (MilliQ H_2_O), which in turn were not statistically different (*p* > 0.05). Samples with Cu concentrations lower than 9 × 10^−6^ g/mL and MilliQ H_2_O all showed viability above 80% (Figure 6).

## 4. Discussion

Living organisms on the microscopic level interact with their environments in complex and often nuanced ways. This is true for bacteria and cells that not only receive nutrition from their environmental media, but also experience toxic stimulation from the same. Titanium-copper alloys have been put forward as a promising antibacterial material [18]. However, the maximum amount of copper that can be used in a biomaterial is correlated to its toxic effect at high concentrations. To elucidate the arena of microorganism interactions with Cu containing environments, the present study aimed to investigate the optimal concentration at which bacterial growth can be deterred while in vitro cyto-toxicity is avoided.

Based on an average American male, the total-body-water can be estimated to be approximately 27 liters [23,34,35] and by assuming a maximum Cu intake of 2.5 mg for a 24-h period [14], the daily-recommended Cu concentration is approximately 5 × 10^−8^ g/mL. The highest concentration used in this study (9 × 10^−3^ g/mL Cu ions in solution) is similar to the Cu ion concentration measured from the almost pure copper alloy (3 × 10^−3^ g/mL), which is much higher than the recommended daily Cu intake (10^5^ times). In fact, a rapid reduction in luminescence was observed immediately after the Cu ions solution was added to the bacteria. This is corroborated by a CFU count of the same bacteria after 5 h, where zero bacterial survival was recorded. Additionally, this concentration induced a similar result in the MC3T3 cells where zero survival of cells was found after 5 and 24 h of exposure. This implies, as expected, that the 9 × 10^−3^ g/mL Cu ion concentration is highly toxic to prokaryotic and eukaryotic cells. These results allow inference that this concentration is ideally avoided for implants of any type that should be in contact with mammalian tissue. Furthermore, the 9 × 10^−4^ g/mL concentration induced a similar response in bacteria and cells and should be viewed in the same cautionary manner, when implant materials are considered. 

A 100-fold dilution from the 9 × 10^−3^ g/mL concentration (9 × 10^−5^ g/mL), however, caused a more gradual bacterial reduction, and the trend in the luminescence recordings over the 7-h test indicated that it could result in bacterial death over a longer period of time. This concentration limited bacterial increase in a nutrient-rich environment, demonstrated by its similar bacterial count to the positive-control after 5 h. Similar Cu ion antibacterial efficacy was reported for *Enterococcus hirae*, where Cu coupons released more than 8 mM (5 × 10^−5^ g/mL) of Cu ions in 30 min [19]. From these results it can be inferred that this concentration can be useful for limiting bacterial growth on implant materials. However, the 9 × 10^−5^ g/mL concentration did cause a significant drop in MC3T3 viability after 5 h (relative to the negative control), and after 24 h of exposure low survival of the MC3T3 cells was observed. This result alludes to the possibility of MC3T3 and bacteria being equally vulnerable to a Cu ion rich environment. This is in line with previous studies on MC3T3-E1 cells [36,37], where it has been found that these pre-osteoblasts are vulnerable to hydrogen peroxide. Consequently, with the addition of Cu ions, the hydroxyl products from the Fenton reaction could be responsible for increased necrosis and apoptosis resulting in low survival. By contrast, prokaryotes of the *Staphylococcus* variety are known to inhibit hydrogen peroxide scavenging, in order to limit hydroxyl production by the Fenton reaction as a means of survival [21]. A Cu ion concentration of 9 × 10^−5^ g/mL did not seem suitable for an optimal cell survival, at least not in static conditions. It is possible that a dynamic and more in vivo-like scenario would yield a more positive result; however, that needs to be verified in further studies.

The survival of MC3T3 cells during the 5- and 24-h test was drastically increased when the Cu ion concentration was reduced from 9 × 10^−5^ to 9 × 10^−6^ g/mL. However, the 9 × 10^−6^ g/mL of Cu ions did not reduce the *Staphylococcus epidermidis* luminescent signal as achieved by the higher Cu ion concentrations. This result is consistent with the counted bacteria from the CFU analysis. Thus, it is reasonable to propose that there could be a Cu ion concentration between 9 × 10^−5^ and 9 × 10^−6^ g/mL, where optimal bacterial reduction and optimal tissue growth can be achieved. For L929 mouse fibroblasts, the lethal dosage (LD_50_) has been determined as 46 × 10^−6^ g/mL of Cu ions [38], which is within the toxicity range determined for the MC3T3 pre-osteoblasts herein. While these studies indicate that MC3T3 cells and *Staphylococcus epidermidis* are sensitive to Cu ion concentrations above 9 × 10^−6^ g/mL, it is possible that the bacteria could develop resistance strategies to Cu ions. Facilitators of this resistance could be a result of the various metal-resistant genes in *Staphylococcus*-type bacteria, such as the copper transporting ATPase *copA* and *mco* (multi-copper oxidase) [39]. 

The concentrations above 9 × 10^−6^ g/mL of Cu ions all showed a viability of the MC3T3 cells similar to the negative control after the same exposure duration. However, these concentrations also gave a bacterial growth that was pronounced and higher than the negative control. In particular, the bacteria number was highest at the 9 × 10^−7^ g/mL using luminescence measurements. Plate counting confirmed that 9 × 10^−7^ g/mL had a high bacterial count, though 9 × 10^−6^ g/mL had the highest average count, however not statistically different from the 9 × 10^−7^ g/mL solution. It can, therefore, be proposed that maximum bacterial growth is stimulated in the range of solutions from 9 × 10^−6^ g/mL to 9 × 10^−7^ g/mL. This result can be understood in light of copper being a stress-inducing ion for *Staphylococcus* bacteria, where studies showed that Cu ion concentrations less than the MIC value of 200 mM resulted in faster growth for bacteria relative to a negative control without copper [21]. This finding sheds light on the complex behavior of bacteria with their surroundings, where the essential co-factor of copper in excess can cause toxicity, but can also cause rapid growth of bacterial populations below the toxic limit. These underlying mechanisms for growth could be elucidated by quantitative polymerase chain reaction (qPCR) and is recommended in further studies. Furthermore, with concentrations lower than 9 × 10^−7^ g/mL, the luminescent signal was reduced and the bacterial count decreased as well. This implies that the 9 × 10^−7^ g/mL is the optimum concentration to induce the highest bacterial growth, and for antibacterial applications this concentration should be avoided. 

The previously studied titanium alloy of 10 wt. % Cu [24], was found to release 7 × 10^−8^ g/mL of Cu ions in 24 h. Therefore, this material will likely release fewer Cu ions per day than 9 × 10^−7^ g/mL, and avoid stimulating optimal growth in *Staphylococcus epidermidis*. This alloy has, however, been proven to have a much larger antibacterial effect with the same bacteria as well as with gram-negative bacteria [18,24] than the one measured with the corresponding Cu ion concentration. The reported antibacterial effect is, therefore, believed to be due to a dual-mechanism of contact killing and ion release [12,19], so that the bacterial population may indeed be effectively reduced by a 10 wt. % Ti-Cu alloy [40]. Further studies are needed to deepen the knowledge about how Cu is killing bacteria.

A limitation to the present study is that only one type of bacteria and cells was investigated. However, the bacterium and cell line investigated herein are both highly relevant. *S. epidermidis* is one of the most prevalent bacteria in implant infections and MC3T3-E1 osteoblasts are one of the most general type of cells investigated in bone models. Nevertheless, the study of Cu ion solutions with other types of bacteria and cell lines, such as *Staphylococcus aureus* and osteoclast precursors, respectively, can further the understanding in this research area and would be of interest to investigate in future studies.

## 5. Conclusions

The findings herein give a deeper view into the behavior of *Staphylococcus epidermidis* and MC3T3 in a Cu ion environment, and indicate that prokaryotes and eukaryotes interact with Cu ions in nuanced ways. The *Staphylococcus epidermidis* and MC3T3 sensitivity was found to be between 9 × 10^−5^ and 9 × 10^−6^ g/mL of Cu ions in solution, where further studies could identify the optimum concentration for prokaryote death and eukaryote survival. The optimal growth of bacteria due to stress responses occurred in the range from 9 × 10^−6^ g/mL to 9 × 10^−7^ g/mL, and is suggested to be avoided in all antibacterial applications. MC3T3 cells, however, showed low survival at Cu concentrations above 9 × 10^−5^ g/mL, requiring further studies in vivo before using materials with such Cu ion release for implantable antibacterial applications.

## Figures and Tables

**Table 1:** Antibacterial Cu ion solutions.

Cu ion Solutions	Sample Solutions									
CuCl_2_·2H_2_0 (g/mL)	0.25 × 10^−1^	0.25 × 10^−2^	0.25 × 10^−3^	0.25 × 10^−4^	0.25 × 10^−5^	0.25 × 10^−6^	0.25 × 10^−7^	0.25 × 10^−8^	0.25 × 10^−9^	0.25 × 10^−10^
Cu ions (g/mL)	9 × 10^−3^	9 × 10^−4^	9 × 10^−5^	9 × 10^−6^	9 × 10^−7^	9 × 10^−8^	9 × 10^−9^	9 × 10^−10^	9 × 10^−11^	9 × 10^−12^

**Table 2:** Cu ion concentration together with standard deviation (SD) and relative standard deviation (RSD) for the measurement made on each token. Note that ‘*’ denotes the thickness of the tokens.

Name	Cu Concentration (mg/L)	Standard Deviation (%)	RSD (%)
99.5% Cu-200um *	2.632	0.0922	3.5
99.5% Cu-200um *	2.902	0.0204	0.7
99.5% Cu-220um *	2.72	0.0006	0.02
TiCu-10-630um *	0.07	0.0012	1.69
TiCu-10-590um *	0.072	0.001	1.41
TiCu-10-525um *	0.063	0.0012	1.94
